# Mutation of the Melastatin-Related Cation Channel, TRPM3, Underlies Inherited Cataract and Glaucoma

**DOI:** 10.1371/journal.pone.0104000

**Published:** 2014-08-04

**Authors:** Thomas M. Bennett, Donna S. Mackay, Carla J. Siegfried, Alan Shiels

**Affiliations:** Department of Ophthalmology and Visual Sciences, Washington University School of Medicine, St. Louis, Missouri, United States of America; Centro de Investigación Príncipe Felipe – CIPF, Spain

## Abstract

Inherited forms of cataract are a clinically important and genetically heterogeneous cause of visual impairment that usually present at an early age with or without systemic and/or other ocular abnormalities. Here we have identified a new locus for inherited cataract and high-tension glaucoma with variable anterior segment defects, and characterized an underlying mutation in the gene coding for transient receptor potential cation channel, subfamily M, member-3 (*TRPM3*, melastatin-2). Genome-wide linkage analysis mapped the ocular disease locus to the pericentric region of human chromosome 9. Whole exome and custom-target next-generation sequencing detected a heterozygous A-to-G transition in exon-3 of *TRPM3* that co-segregated with disease. As a consequence of alternative splicing this missense mutation was predicted to result in the substitution of isoleucine-to-methionine at codon 65 (c.195A>G; p.I65 M) of TRPM3 transcript variant 9, and at codon 8 (c.24A>G; p.I8 M) of a novel TRPM3 transcript variant expressed in human lens. In both transcript variants the I-to-M substitution was predicted *in silico* to exert damaging effects on protein function. Furthermore, transient expression studies of a recombinant TRPM3-GFP reporter product predicted that the I-to-M substitution introduced an alternative translation start-site located 89 codons upstream from the native initiator methionine found in eight other TRPM3 transcript variants (1–8). Collectively, these studies have provided the first evidence that *TRPM3* is associated with inherited ocular disease in humans, and further provide support for the important role of this cation channel in normal eye development.

## Introduction

Mendelian forms of cataract constitute a clinically and genetically heterogeneous disorder of the ocular lens that occurs with an estimated prevalence of 1–3 cases per 10,000 births [1], [2]. Typically, inherited cataract presents with an early onset ranging from birth (congenital) or infancy (infantile) into the fourth decade and, under slit-lamp examination, exhibits considerable phenotypic variation with respect to the location, shape, density and progression of opacities within the lens [3]. Congenital and infantile forms of cataract are a clinically important cause of impaired form-vision development (deprivation amblyopia) that despite surgical intervention can pose a lifelong risk of progressive visual impairment, notably as a result of glaucoma [4]–[6]. Cataract may be inherited either as an isolated lens phenotype, often with autosomal dominant transmission and high penetrance, or as part of many genetic syndromes and metabolic disorders (OMIM.org) involving systemic abnormalities (e.g. galactosemia, MIM230400) and/or other ocular defects (e.g. anterior segment dysgenesis, MIM107250). So far, genetic studies have identified over 50 genes and loci for inherited cataract, with or without other ocular signs, including those for α-crystallins, β/γ-crystallins, α-connexins and other lens membrane or cytoskeleton proteins, several transcription factors (e.g. *HSF4*, *PITX3*, *MAF*), and an increasing spectrum of functionally diverse genes (e.g. *EPHA2*, *CHMP4B*, *TDRD7*, *FYCO1*) [2], [7]. Consequently, no clear genotype-phenotype correlation has been observed for inherited cataract rendering both clinical classification and molecular diagnosis challenging.

Transient receptor potential (TRP) channel genes encode a superfamily of variably selective cation influx channels that function as polymodal cellular sensors in diverse physiological processes including the perception of light, temperature, pressure and pain [8]–[10]. The TRP superfamily derives its name from the visually impaired *trp*-mutant of *Drosophila* that was found to exhibit a transient photoreceptor response to steady light rather than the sustained electroretinogram observed in wild-type flies [11]. In mammals, 28 TRP channel genes have been identified, and based on sequence similarity they are divided into six sub-families, namely; canonical (TRPC1–7), vanilloid (TRPV1–6), melastatin (TRPM1–8), ankyrin (TRPA1), polycystin (TRPP1–3), and mucolipin (TRPML1–3) [9]. All TRP proteins share a predicted transmembrane topology with six-transmembrane domains including the channel pore-forming loop located between the 5^th^ and 6^th^ domains. The cytosolic N- and C- termini are variable in length and contain different combinations of functional sub-domains including; N-terminal ankyrin repeats, calmodulin-binding motifs, TRP-boxes, coiled-coil regions, and C-terminal enzyme domains [12]. Functional TRP channels are assembled from tetramers of identical (homomeric) or similar (heteromeric) TRP subunits, and exhibit highly variable permeability to Ca^2+^ and several other monovalent and divalent cations [9]. TRP channels exhibit wide tissue distribution in both plasma-membranes and intracellular membranes particularly from brain and kidney [13]–[14].

So far mutations in at least ten TRP genes spanning all sub-families have been associated with over a dozen human genetic diseases, referred to as ‘channelopathies’ [15]–[17]. These include skeletal dysplasias, sensory neuropathies and spinal muscular atrophies (*TRPV4*, MIM 605427), kidney diseases (*TRPC6*, MIM 603652; *TRPP1*, MIM 601313), mucolipidosis type-IV (*TRPML1*, MIM 605248), familial episodic pain syndrome (*TRPA1*, MIM 604775), and Olmsted syndrome (*TRPV3*, MIM 607066). Within the melastatin sub-family, mutations have been implicated in Guamanian amyotropic lateral sclerosis-parkinsonism/dementia (*TRPM7*, MIM 605692), progressive familial heart block type-1B (*TRPM4*; MIM 604559), hypomagnesemia with secondary hypocalcemia (*TRPM6*; MIM 602014), and congenital stationary night blindness (CSNB) in humans (*TRPM1*, MIM 603576) and in horses homozygous for *Leopard Complex* (*LP*) coat spotting [18]–[22]. *TRPM3* (melastatin-2), which is most phylogenetically conserved with *TRPM1* (melastatin-1), has not been unambiguously linked with inherited human disease. Rare deletions involving *TRPM3* have been reported in cases of Kabuki syndrome and autism, whereas, common non-coding variants in *TRPM3* have been tentatively associated with longevity and elevated levels of low-density lipoprotein cholesterol and triglycerides [23]–[26].

Here we map, identify, and characterize a mutation in *TRPM3* associated with inherited cataract and glaucoma linked to human chromosome 9q.

## Results

### A new locus for cataract and glaucoma maps to human chromosome 9

**Figure 1:**
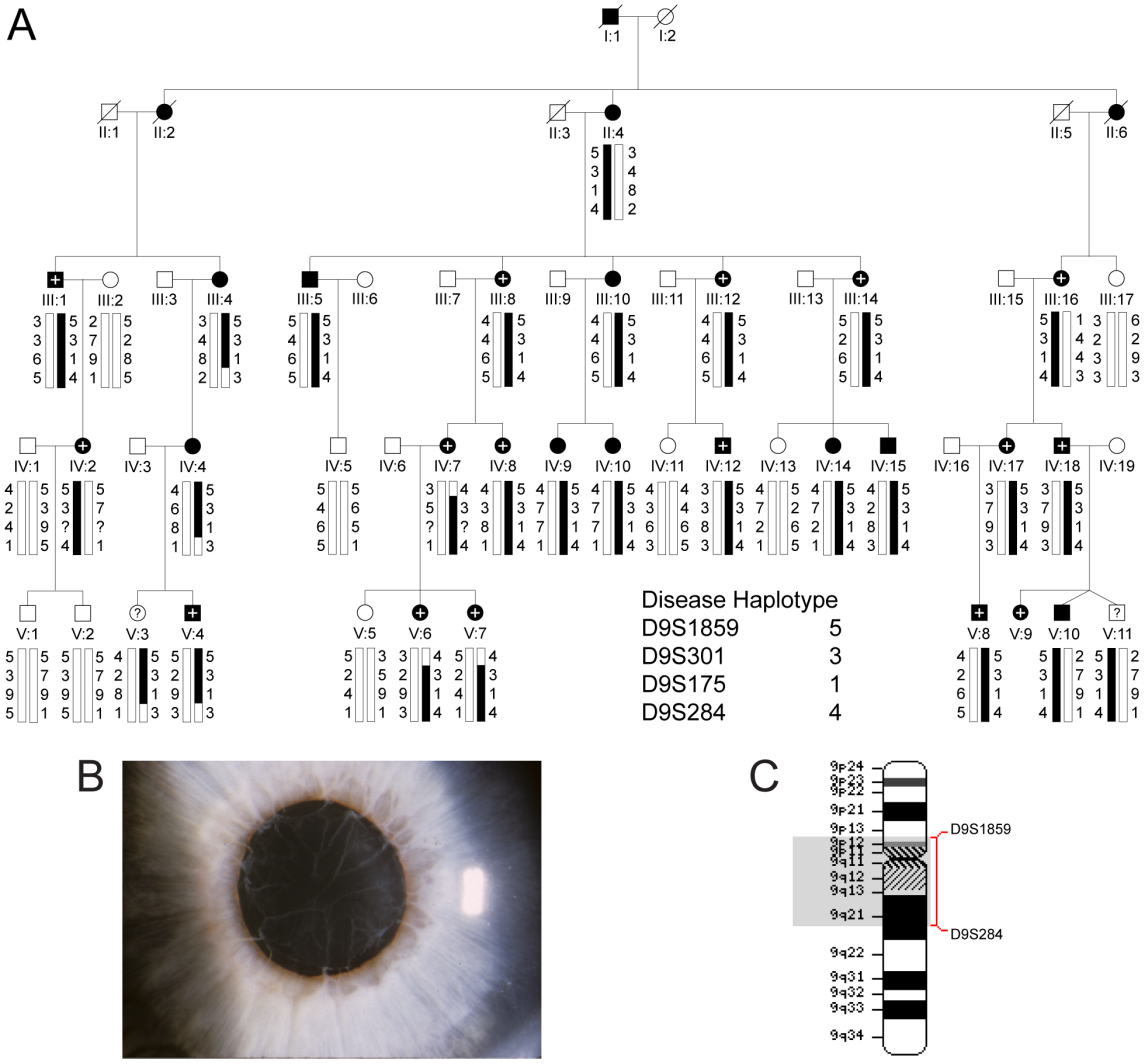
Linkage analysis of autosomal dominant cataract and glaucoma co-segregating in a 5-generation family. (**A**) Pedigree and haplotype analysis showing segregation of markers across chromosome 9cen, listed in descending order from the short-arm telomere (9p-tel). Affected males (filled squares) and females (filled circles) that were diagnosed with cataract and glaucoma are indicated with a + symbol. (**B**) Ocular phenotype of affected individual V:4 presenting with high-tension glaucoma and persistent pupillary membrane characterized by fibrous strands extending from the iris border across the pupil. (**C**) Partial ideogram of chromosome 9 showing cytogenetic location of the ocular disease locus. Note, cytogenetic band regions p11.1, q11 and much of q12 are gene-poor centromeric regions, flanked by gene-rich regions in bands p11.2–p13.3 on the short arm and q13–q21.31 on the long arm.

We investigated a 5-generation Caucasian-American pedigree mostly from the mid-west United States segregating cataract and glaucoma with autosomal dominant transmission (Figure 1a; Table S1). Review of ophthalmic records indicated that the cataract was usually bilateral and generally described as congenital, infantile or developmental, with age-at-diagnosis varying from birth (congenital) to 35 years, and age-at-surgery in the first eye ranging from 4 to 40 years. No slit-lamp images of the cataract were available; however, in affected individual IV:17 from the pedigree opacities were described as punctate cortical, and in affected individual V:4 posterior sub-capsular opacities were documented by slit-lamp examination in the medical record. All of the family members with cataract had visually significant lens opacities affecting best corrected visual acuity and underwent lens extraction. Of the 25 relatives with cataract, 15 (60%) were also diagnosed with glaucoma. Glaucoma diagnosis was supported by significantly elevated intraocular pressure (IOP) >30 mm Hg and, in some cases, consistent visual field and/or optic nerve abnormalities. We note that our records of current glaucoma status for two of the undiagnosed individuals (V:10, V:11) are incomplete due to unsuccessful follow-up attempts. Age-at-diagnosis for glaucoma ranged from birth into the fifth decade, and age-at surgery when performed varied from 11-30 years. Glaucoma was variously described in four individuals (III:16, IV:18, V:4, V:9) as congenital, infantile, juvenile, or developmental open-angle by gonioscopic findings. However, in one affected individual (III:1) chronic angle-closure glaucoma was suspected, and in two others (III:14, IV:2) secondary glaucoma was suspected. Typically, cataract was diagnosed before high-tension glaucoma (IOP>30 mm Hg). However, in one case (III:1) glaucoma was diagnosed before cataract, and one individual (III:16) was diagnosed simultaneously with congenital cataract and glaucoma.

In addition to cataract and glaucoma several other ocular findings were reported in certain family members (Table S1). Bilateral persistent pupillary membrane (PPM) was diagnosed in affected male V:4 (Fig. 1b) and his older sister (V:3). However, the latter has not been diagnosed with cataract or glaucoma at age 24 years raising the possibility of incomplete penetrance. Retinal detachment was reported in at least three affected individuals (IV:2, IV:18, V:9), and in one case (V:10) retinopathy of prematurity (ROP) was documented. However, the ROP is likely to be a coincidental finding. Finally, in one individual (V:9), who was unavailable for genetic testing, the ocular spectrum included suspected infantile glaucoma, bilateral Haab's striae (horizontal breaks in Descemet's membrane), anterior segment dysgenesis, megalocornea, mild corectopia, and PPM. However, intra-ocular pressure (IOP) and fundus (disc) were normal, and cataract was absent at eight years of age. Overall, while early-onset cataract and atypical high-tension glaucoma were commonly observed in this family, other ocular abnormalities that particularly affect the anterior segment may co-exist.

For genome-wide linkage analysis 24 consenting members of the family (19 affected and 5 unaffected) were genotyped by means of a linkage panel comprising 6090 SNP markers uniformly spaced at an average genetic distance of 0.58 cM. Using parametric two-point analysis we detected significant evidence of linkage at SNP markers rs1333342 (pLOD = 4.53), rs735914 (pLOD = 3.72), and rs713478 (pLOD = 3.45) located on chromosome 9q (Figure S1a). Similarly, parametric multipoint analysis detected strong evidence of linkage between SNP markers rs2031197-(∼333.4 kb)-rs1333342 (pLOD = 3.31) on 9q (Figure S1b). Haplotype analysis of the pedigree further identified a common disease interval spanning the pericentric region (9cen), rs987187-(∼46 Mb)-rs2378592 (chr9: 38,375,215-84,495,227 bp) that co-segregated with the disease in all affected relatives (Table S2).

To validate the SNP disease interval from the genome-wide linkage scan, all 36 relatives were re-genotyped with microsatellite markers. We obtained strong confirmation of linkage to 9q (Table 1) using markers D9S175 (Z_max_ = 8.05, θ_max_ = 0), D9S301 (Z_max_ = 7.59, θ_max_ = 0), and D9S1876 (Z_max_ = 6.42, θ_max_ = 0). Haplotype analysis detected an affected female (III:4) and three of her descendents (IV:4, V:3, V:4) who were recombinant at D9S284, and three affected females (IV:7, V:6, V:7) who were recombinant at D9S1859 (Figure 1a). However, no further obligate recombinants were detected at seven intervening markers (Table 1), placing the disease locus in the reduced physical interval D9S1859-(∼41.5 Mb)-D9S284.

**Table 1:** Two-point Lod scores (Z) for linkage between the ocular disease locus and markers on chromosome 9 listed in physical distance (Mb) from the short-arm telomere (9p-tel).

Marker (recombinant)	Mb	Z at recombination fraction (θ) =							
		0.00	0.05	0.10	0.20	0.30	0.40	Z_Max_	Θ_Max_
D9S1804	35.96	-∞	3.98	3.79	3.00	1.99	0.88	3.98	0.05
D9S1859 (IV:7, V:6, V:7)	36.63	-∞	4.11	3.94	3.17	2.14	0.99	4.11	0.05
D9S1874	37.22	6.76	6.13	5.47	4.08	2.62	1.17	6.76	0.00
D9S2148	38.30	5.18	4.76	4.27	3.19	1.99	0.81	5.18	0.00
rs2073478 (V:6)	38.40	-∞	0.16	0.32	0.34	0.26	0.14	0.36	0.16
*TRPM3* (c.195A>G)	73.48	9.51	8.75	7.96	6.23	4.30	2.14	9.51	0.00
D9S301	73.80	7.59	6.91	6.19	4.67	3.04	1.35	7.59	0.00
D9S1876	75.23	6.42	5.86	5.28	4.07	2.78	1.44	6.42	0.00
D9S175	77.95	8.05	7.37	6.65	5.12	3.48	1.73	8.05	0.00
D9S284 (III:4)	78.09	-∞	6.37	5.94	4.68	3.20	1.60	6.44	0.03
D9S276	78.51	-∞	4.45	4.19	3.28	2.14	0.89	4.46	0.04
D9S1122	79.69	-∞	2.39	2.69	2.52	1.93	1.05	2.71	0.12
D9S1123	80.42	-∞	2.84	2.77	2.29	1.63	0.87	2.84	0.06

To further refine the disease interval we have genotyped the existing critical recombinant individuals (III:4, IV:4, IV:7, V:3, V:4, V:6, V:7) with additional SNP markers located within the boundaries of the microsatellite interval using conventional Sanger sequencing. Critical affected individual V:6 was also found to be recombinant at SNP marker rs2073478 (G/T), which is located within *ALDH1B1* approximately 1.77 Mb centromeric to D9S1859 (Table 1). However, no further recombinants were detected at more centromeric SNP markers indicating that the disease locus lay in the refined physical interval rs2073478-(∼39.7 Mb)-D9S284 (chr9: 38,396,065-78,086,081 bp).

Our disease interval spans the centromere and excludes the adjacent locus for autosomal recessive adult-onset progressive pulverulent cataract (CTPL1/CAAR) that lies in the interval D9S1123-(∼10 Mb)-D9S257 on 9q [27], [28]. In addition the gene coding for Tudor domain containing-7 (*TDRD7*, Gene ID: 23424), which has recently been associated with autosomal recessive cataract [29], lies approximately 20 Mb telomeric (chr9: 100,174,302-100,258,407 bp) to the disease interval and was excluded as the causative gene.

### Exome and custom-target sequencing identifies a novel missense mutation in TRPM3

According to the NCBI Map Viewer database (Build 37.3) the refined disease interval contained over 270 positional candidate genes, none of which had been previously associated with eye disease in humans. In order to scan genes in the interval for mutations we undertook whole-exome and custom-target next-generation sequencing of an affected individual (III:16) and an affected-parent-child-unaffected-sibling trio (IV:7, V:5, V:6), respectively, from the pedigree. For exome sequencing (III:16), 96.2% of total paired-end reads (133,419,338) were mapped to the genome, and 78% of mapped reads were present in the captured exome, of which 95.5% achieved a coverage ≥5X. Similarly, for custom-target sequencing (IV:7, V:5, V:6), >98% of total reads (average 22,175,122) were mapped to the target interval on chromosome 9, and >61% of mapped reads were present in the captured target of which >98% achieved a coverage of ≥5X with no unexpected gaps.

Combined, both strategies identified the same list of four heterozygous, exonic variants (three non-synonymous and one frame-shift deletion) within the disease interval in all three index affected relatives (III:16, IV:7, V:6) that were not present in the unaffected relative (V:5) (Table 2). Of these variants, three had known reference sequence (rs) numbers cited in the dbSNP or 1000Genomes databases with no known clinical significance reported. The minor allele frequency (MAF) of two of the non-synonymous variants, rs143826416 (*FAM75A3*) and rs41310055 (*GDA*), were 43.4% and 1.7%, respectively. Both variants were predicted to be benign using PolyPhen-2, and rs41310055 (*GDA*) was also present on the Exome Variant Server (EVS) (http://evs.gs.washington.edu/EVS). No MAF was retrieved for the frame-shift variant, rs200487787 (*CNTNAP3B*), and it was not present on the EVS. However, Sanger sequencing confirmed that the frame-shift variant did not co-segregate with disease in the pedigree (data not shown). Taken overall, these observations effectively exclude three of the four variants as causative for disease.

**Table 2:** Sequencing variants detected in affected but not unaffected members of the family within the ocular disease interval on chromosome 9 (Figure 1C).

Chr	Position (bp)	Gene Region	Gene Symbol	Nucleotide Variant	Protein Variant	Affected + Variant	Unaffected + Variant	Translation Impact	SIFT Prediction (Score)	PolyPhen-2 Prediction (Score)	dbSNP ID	MAF (%)	Found on EVS
9	43,62,5382	Exonic	*FAM75A3*	c.3305C>T	p.P1102L	3	0	Nonsynonymous	Tolerated (0.06)	Benign (0.395)	rs143826416	43.4	No
9	43,844,265	Exonic	*CNTNAP3B*	c.1599delG	p.L534fs*	3	0	Frameshift			rs200487787	None	No
9	73,479,410	Exonic; 5′UTR	*TRPM3*	c.195A>G	p.I65M	3	0	Nonsynonymous	Intolerant (0.00)	Probably Damaging (1.000)	Novel	None	No
9	74,865,703	Exonic; 3′UTR	*GDA*	c.1399C>T	p.P467S	3	0	Nonsynonymous	Intolerant (0.00)	Benign (0.000)	rs41310055	1.7	Yes

**Figure 2:**
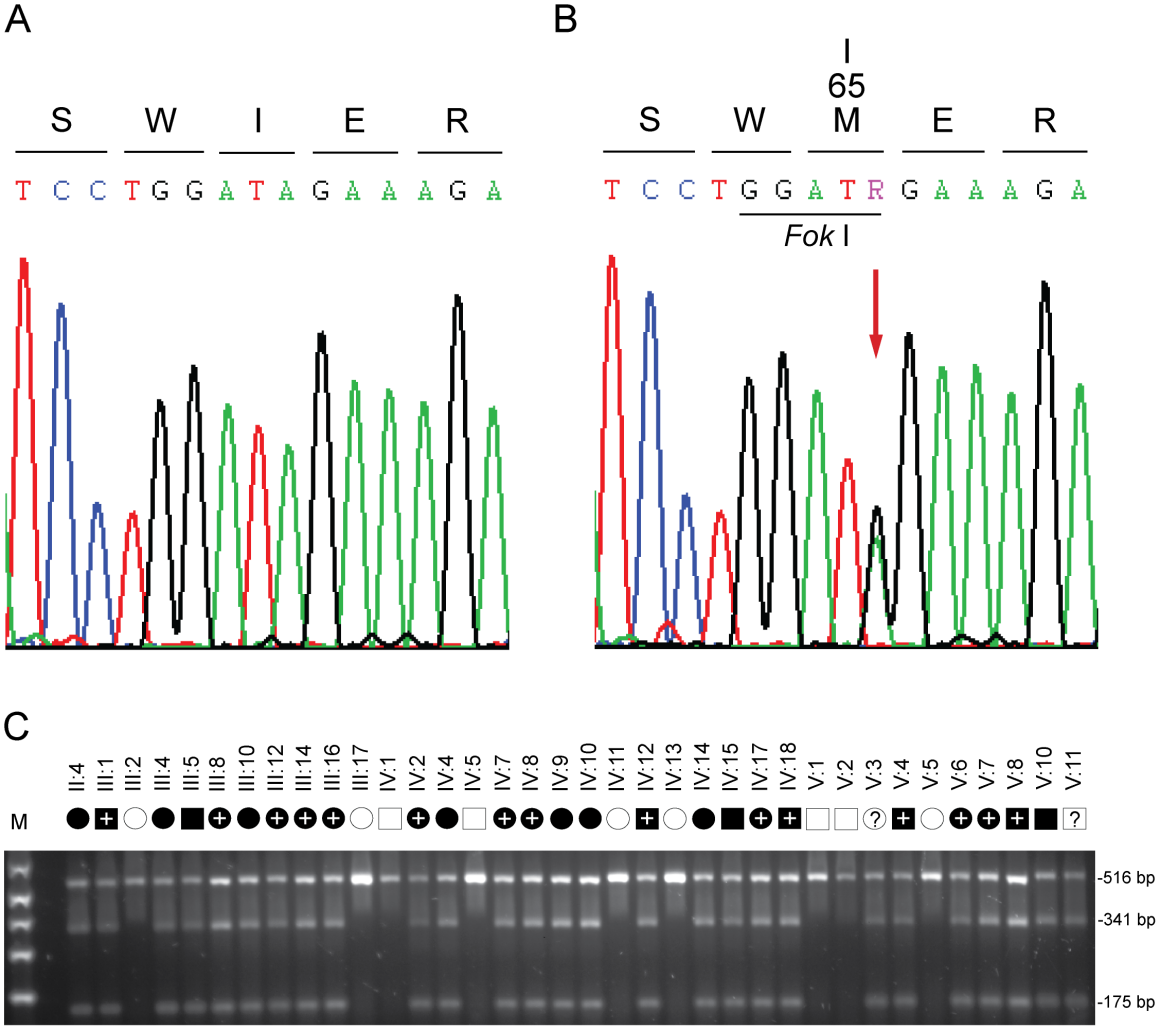
Mutation analysis of *TRPM3*. (**A**) Sanger sequence trace of the wild-type allele showing translation of isoleucine (I) at codon 65 (ATA) of variant 1 (isoform k), and at codon 8 of a novel lens variant. (**B**) Sequence trace of the mutant allele showing the heterozygous A-to-G transition (denoted R by the International Union of Pure and Applied Chemistry [IUPAC] code) that is predicted to result in the missense substitution of methionine (ATG) for isoleucine at codon 65 (c.195A>G, p.I65 M) of isoform k, and at codon 8 of a lens abundant isoform (c.24A>G, p.I8 M). (**C**) Restriction fragment length analysis showing gain of a *Fok* I site (5′GGATG[9/13]) that co-segregated with affected individuals heterozygous for the A-to-G transition (175 bp).

The remaining non-synonymous variant was novel, and comprised a heterozygous A>G transition that introduced a *Fok* 1 restriction site located in exon-3 of the gene coding for transient receptor potential cation channel subfamily M, member-3 (*TRPM3*). This missense change occurred at nucleotide position 195 from the first base (A) of the translation start codon in the cDNA sequence for *TRPM3* transcript variant-9 (c.195A>G). Sanger sequencing confirmed the presence of the c.195A>G change in exon-3 from the three index affected relatives (Figure 2b), and excluded mutations in other exons and splice-sites of *TRPM3*. Allele-specific PCR amplification and *Fok* 1 restriction fragment length analysis confirmed that the c.195A>G change co-segregated with affected but not unaffected relatives across the family (Figure 2c). Moreover, when we tested the c.195A>G change as a bi-allelic marker with a notional frequency of 1% in a two-point LOD score analysis we obtained highly significant confirmation of linkage to *TRPM3* (Z_max_ = 9.51, θ_max_ = 0.0) (Table 1). Finally, this variant was not detected in a panel of 192 normal unrelated Caucasian individuals (384 chromosomes) using allele-specific *Fok* 1 restriction fragment analysis described in Figure 2c (data not shown). Combined, our sequence and genotype data strongly suggest that the c.195A>G transition constituted a causative mutation and not a benign variant in linkage disequilibrium with the disease.

### TRPM3 generates a novel transcript in human lens, and gains an alternative translation start-site in other TRPM3 transcript variants

**Figure 3:**
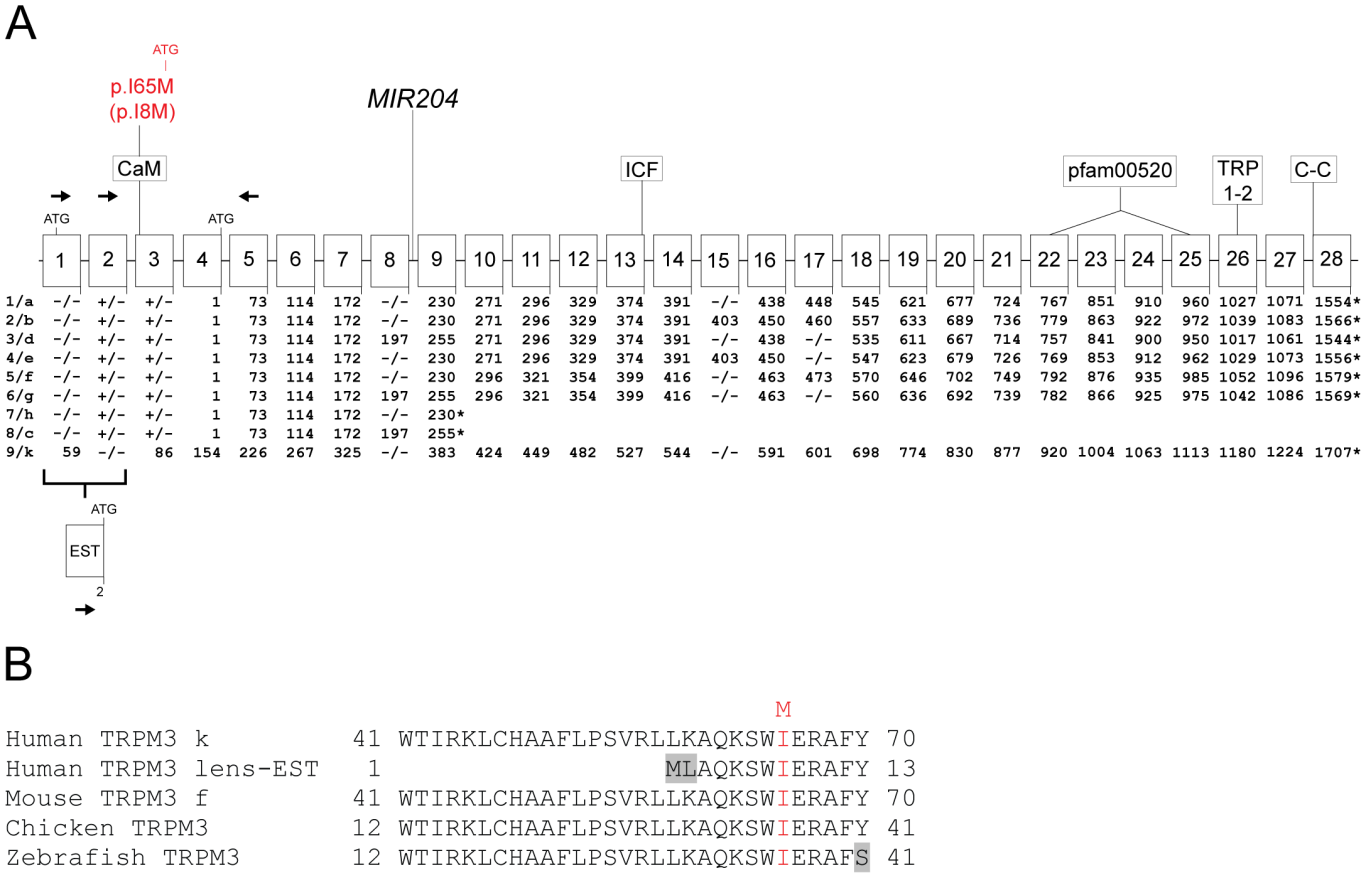
Schematic showing the gene structure of TRPM3. (**A**) Exon organization, splice variants and protein domains. Exons are indicated by numbered boxes (1–28), and codon numbers are shown below each coding exon. Translation start and stop sites are denoted by ATG and asterisks, respectively. Transcript variants are numbered (1–9), and corresponding protein isoforms are indicated by letters (a–h, k). Exons subject to alternative splicing (1, 2, 8, 15 and 17) are indicated by plus and minus symbols below each exon. (−/−) indicates that the exon is absent from both the transcript variant and protein isoform. (+/−) indicates that the exon is present in the transcript variant but is not translated. Exon 2, exon 8 and exon 15 are skipped in transcript variant 9 (isoform k). In transcript variants 1–8 (isoforms a–h) exon 2, exon 3 and all of exon 4 except the last 3 bases (ATG) are non-coding. EST denotes an expressed sequence tag (BM712132) that replaces exon 1 of variant 9, and exon 2 of variants 1–8, joining directly to exon 3 in a novel lens abundant transcript variant (KF987075). Arrows indicate the orientation and exon location of PCR primers used to amplify and sequence the 5′-ends of TRPM3 transcripts. The micro-RNA gene, *MIR204*, is located in intron 8. The predicted I-to-M substitution (red) identified in the family studied here is located in a putative calmodulin-binding (CaM) motif [31] near the N-terminus of isoform k and the novel lens isoform. The approximate locations of conserved protein domains are indicated: (pfam00520) ion-transport domain; (TRP1–2) transient receptor potential box 1 and 2; (C–C) coiled-coil domain, and (ICF) amino-acid sequence indispensible for channel function [45]. Note that variants 7 and 8 encode short isoforms h and c, respectively, lacking the conserved domains above. These short cytoplasmic isoforms may play a role in regulation of the full-length transmembrane isoforms [64]. (**B**) Amino acid sequence alignment of the N-terminal calmodulin-binding motif of human TRPM3 isoform-k, a novel human lens isoform, and homologs from other species showing conservation of isoleucine at the site of the predicted methionine substitution (red). Divergent amino-acid residues are shaded grey.

**Figure 4:**
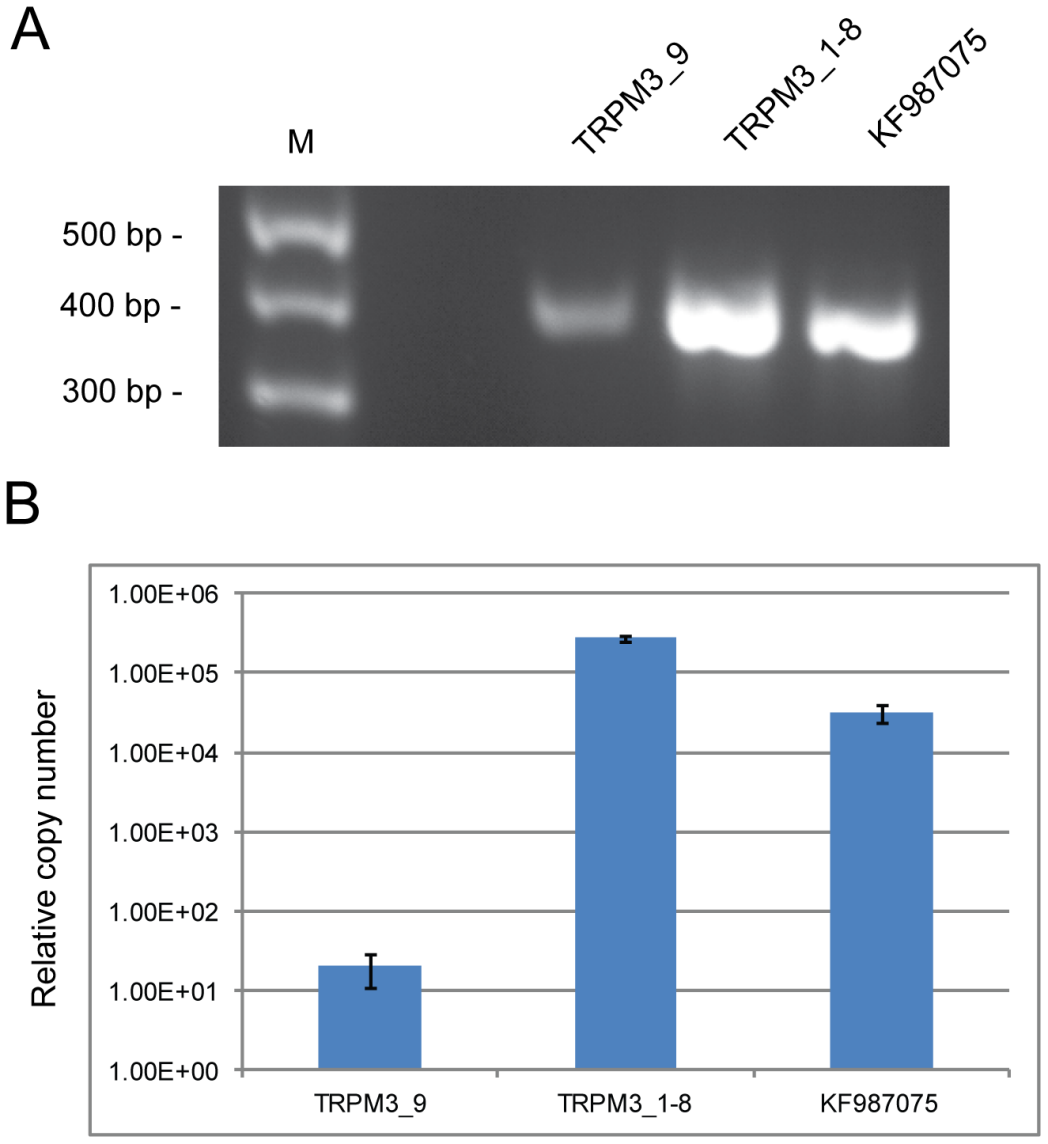
Alternative splicing of *TRPM3* transcripts in the human lens. (**A**) RT-PCR showing presence of transcripts for variant 9 (exon 1–5, 411 bp), variants 1–8 (exon 2–5, 414 bp) and a novel lens variant, KF987075 (EST - exon 5, 403 bp). (**B**) Quantitative RT-PCR showing relative copy numbers of TRPM3 variant 9 (2.06E+01, SD 9.46E+00), variants 1–8 combined (2.76E+05, SD 2.15E+04), and lens variant KF987075 (3.17E+04, SD 8.07E+03) standardized against RPL19. TRPM3 primers used for amplification were as follows; RT1F - RT5R for variant 9, RT2F - RT5R for variants 1–8, and ESTF - RT5R for lens variant KF987075 (Table S5).

The reference sequence for *TRPM3* (Gene ID: 80036) comprises 28 exons that generate at least nine transcript variants (1–9) encoding nine protein isoforms (a–h, k) by means of alternative splicing (Figure 3, Table S3). In order to profile TRPM3 transcript variants expressed in the human lens we undertook reverse transcript PCR (RT-PCR) and 5-prime rapid amplification of cDNA ends (5′-RACE). In postmortem lens RNA we detected strong evidence for multiple TRPM3 reference transcripts (Figure 4). These included transcript variant-9, which encodes protein isoform-k, and some or all of transcript variants 1–8, which encode protein isoforms a-h (Table S3). In addition, we detected abundant levels of a novel transcript variant (GenBank accession number: KF987075) that substituted exon-1 of variant-9 with an expressed sequence tag (EST, GenBank accession number: BM712132) previously found in human lens ([30], Figure 4, Figure S2).

The c.195A>G missense change in TRPM3 transcript variant-9 identified above occurred at the third base position of codon 65 (ATA>ATG), and was predicted to result in the conservative substitution of isoleucine-to-methionine (p. I65 M) in TRPM3 isoform-k at the level of protein translation. Notably in the lens transcript variant, which encoded a truncated N-terminal domain compared with isoform-k, the missense change occurred at codon 8 (c.24A>G) shifting the predicted amino-acid substitution accordingly (p.I8M). The predicted I-to-M substitution represented a relatively conservative amino acid change with the non-polar side-group of isoleucine (-C_4_H_9_) replaced by the non-polar sulfur-containing side-group of methionine (-C_2_H_4_-S-CH_3_). However, cross-species amino-acid alignment of TRPM3 confirmed that the substituted isoleucine residue is evolutionarily conserved in bony vertebrates, and is located within a putative calmodulin-binding motif near the N-terminus (Figure 3, [31]). Further, *in silico* missense mutation prediction analysis using the SIFT and PolyPhen-2 algorithms were consistent with damaging effects on protein function (Table 2).

**Figure 5:**
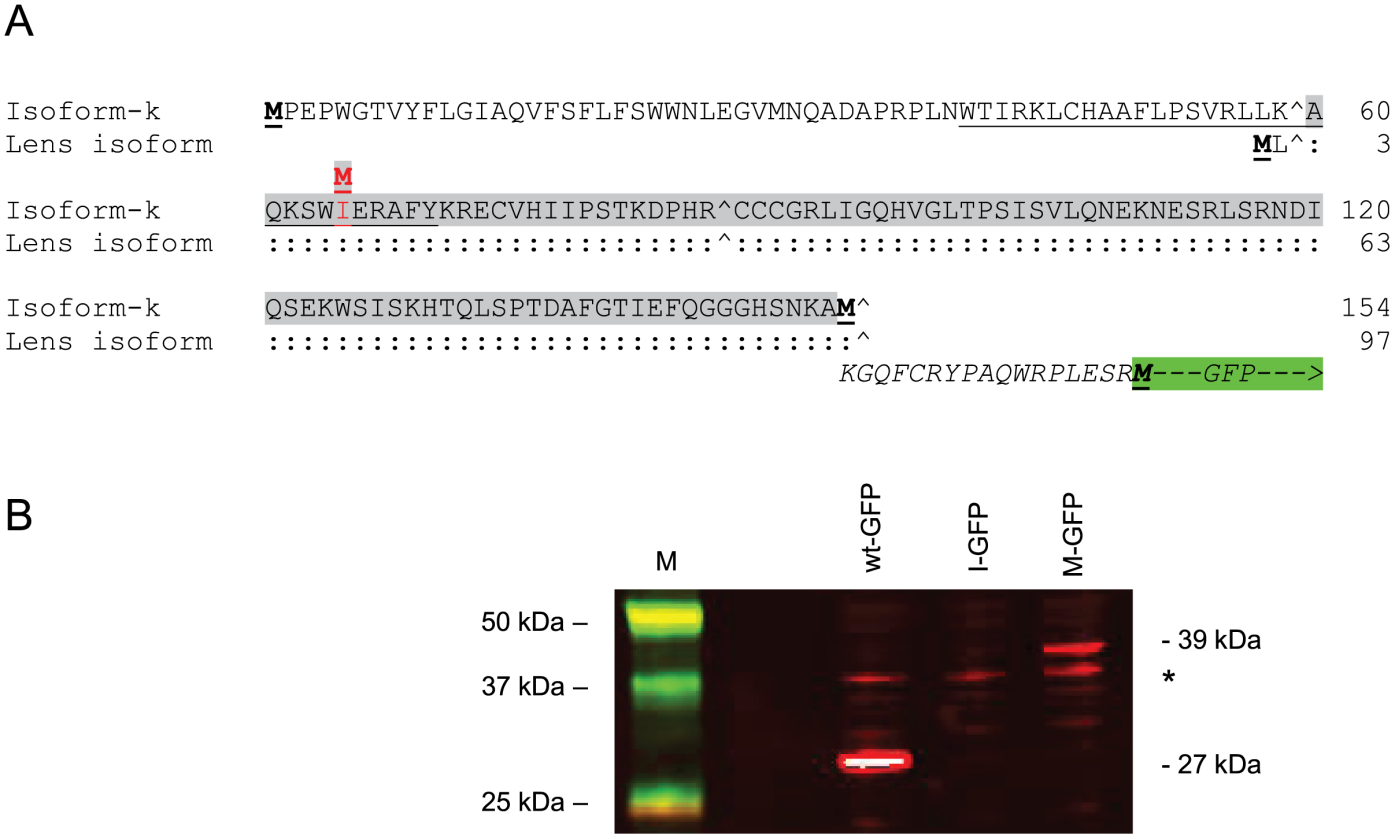
Transient expression of a TRPM3-GFP fusion product in HEK293T cells. (**A**) N-terminal amino-acid sequence of TRPM3 isoform k (NP_001007472), and a lens abundant isoform (KF987075). The sequence coding for amino-acids 60-153 (exon 3) of isoform k (codons 3–96 of the lens isoform) that was inserted into the GFP fusion vector is shaded grey. The p.I65M substitution in isoform k (p.I8M in the lens isoform) is shown in red. Translation initiator methionine residues are shown in bold and underlined. The translation start-site for isoform k is located 57 codons upstream of that for the lens isoform, and 153 codons upstream of that for isoforms 1–8. Italics denote the 17 amino-acid sequence linking the TRPM3 sequence in-frame to GFP (green box). The GFP translation start-site is located in-frame 106 codons downstream from the I-to-M substitution in isoform k and the lens isoform. (:) denotes identical amino-acids, (∧) indicates intron boundaries between exons 1, 3 and 4 (exon 2 is skipped). The CaM binding domain is underlined. (**B**) Immunoblot analysis of transfected HEK293T cell lysates showing presence of the predicted TRPM3-GFP fusion product (∼39 kDa) that is ∼12 kDa larger than native GFP (27 kDa). (*) indicates non-specific cross-reaction with the HEK293T cell lysate.

In human TRPM3 transcript variant-9, the translation start codon is located in exon-1, and the c.195A>G (p.I65M) change in exon-3 (Figure 3). Similarly, in the lens transcript variant the start codon is located in the EST and the c.24A>G change (p.I8M) is located in exon-3. By contrast, in human TRPM3 transcript variants 1–8 the translation start-codon is located at the end of exon-4 (Figure 3) effectively placing the predicted p.I65M and p.I8M substitutions in the 5′-untranslated region (5′-UTR) in-frame with the native start-codon lying downstream. Interestingly, the predicted I-to-M substitution is embedded in a partially consensus Kozak sequence raising the possibility that it functions as a novel translation start-site (Figure 5a, Figure S3). In order to test this possibility we sub-cloned part of the 5′-coding sequence from variant-9 (codons 60–153) into a GFP fusion vector such that the I-to-M substitution was in-frame with the consensus translation start-site for GFP located downstream (Figure 5a, Figure S3). Following transient expression of the mutant (methionine) sequence in HEK293T cells and immunoblot analysis with GFP antibody, we detected a soluble antigen (Mr ∼39 kDa) that was ∼12 kDa larger than the native GFP (∼27 kDa, Figure 5b). This larger antigen was consistent with translation of a TRPM3-GFP fusion protein starting from the I-to-M substitution site. By contrast, the ‘wild-type’ (isoleucine) sequence did not produce a detectable GFP-fusion product (Figure 5b) most likely because several out-of-frame translation start-codons were present in the TRPM3 sequence between the I-to-M substitution site and the translation start-site for GFP (Figure S3). These data predict that, in heterozygous individuals, the I-to-M change introduces an alternative translation start-site in transcript variants 1–8, which is able to compete with the endogenous start-sites potentially resulting in the addition of 89 amino acid residues to the N-terminal domain of protein isoforms a–h.

## Discussion

In cases where early-onset cataract and glaucoma are co-inherited, suspicion is first directed toward genes encoding ocular transcription factors, notably *PAX6, FOXE3, PITX3*, and *MAF* that control development of anterior ocular structures including the cornea, iris, ciliary-body, iridocorneal angle drainage system (trabecular meshwork and Schlemms canal) and lens [32]–[34]. Following exclusion of these and many other candidate genes we have identified *TRPM3* on human chromosome 9q as a novel gene for autosomal dominant cataract and high-tension glaucoma. The A-to-G mutation discovered in transcript variant-9 (c.195A>G) was predicted to result in a missense substitution (p.I65M) in TRPM3 isoform-k that was located within a putative calmodulin binding motif. However, because of extensive alternative splicing of TRPM3 transcripts, the A-to-G mutation was also predicted to cause a missense substitution (c.24A>G; p.I8M) in a novel TRPM3 transcript abundant in human lens. In both transcript variants, the I-to-M substitution was predicted *in silico* to exert damaging effects on protein function. Moreover, the I-to-M substitution was predicted *in vitro* to function as an alternate translation initiation-site in TRPM3 transcript variants 1–8 resulting in aberrant N-terminal extension of TRPM3 isoforms a–h. Structure-function analyses of TRPM channels indicates that the N-terminal and C-terminal domains form a large cytoplasmic complex that functions in membrane localization, tetrameric assembly, and ligand interactions [35], [36]. Conceivably, abnormal extension of the N-terminal domain may reduce overall protein stability and compromise some or all of these functions. While we cannot exclude loss-of-function defects, these observations raise the likelihood that the I-to-M substitution triggers deleterious gain-of-function effects leading to lens opacities, high-tension glaucoma, and variable anterior segment defects.

In mice, both *Trpm3* and its intronically hosted, non-coding micro-RNA gene (*Mir204*) are co-expressed in ocular tissues, particularly the ciliary-body, lens epithelium, and retinal pigment epithelium [37]–[39]. Recently, the paired-box transcription factor gene, *Pax6*, has been shown to co-regulate *Trpm3* and *Mir204* during mouse eye development, and an evolutionarily conserved mechanism has been described in medaka fish [40]–[42]. Up-regulation of miR-204 results in suppression of several target genes involved in neurogenesis, cell-motility, and epithelial-to-mesenchymal transition (EMT), consistent with a role in determining and/or maintaining epithelial cell fate [43], [44]. By contrast, the precise function(s) of *Trpm3* up-regulation in ocular tissues remains unclear. TRPM3 has been shown to function *in vitro* as a spontaneous, steroid-activated, Ca^2+^-permeable channel, with selectivity for other cations influenced by alternative splicing [45], [46]. *In vivo*, TRPM3 channel activity has been found to facilitate diverse cellular processes including, insulin release by pancreatic β-cells [47], mechano-sensing in vascular smooth muscle cells [48], and thermo-sensing by dorsal root ganglia neurons [49]. Recently, *Trpm3*-null mice were found to display an attenuated pupillary light reflex (iris constriction) under bright light and dim light conditions [50]. The abundant expression of *Trpm3* in the ciliary body coupled with the requirement for sustained Ca^2+^ influx during ciliary muscle contraction, support the notion that *Trpm3* functions in regulating pupil constriction [50]. In addition to pupil response, changes in the contractile state of the ciliary muscle are known to modulate aqueous humor outflow via the trabecular meshwork [51]. Further, the ciliary epithelium (pigmented and non-pigmented) co-operate in the production of aqueous humor [52]. These observations raise the possibility that *Trpm3* expression in the ciliary body may influence intra-ocular pressure. Outside the ciliary body, the abundant expression of *Trpm3* in the lens epithelium and retinal pigment epithelium suggests that this cation channel may also participate in the maintenance of intracellular Ca^2+^ essential for preserving lens transparency, and in the regulation of Ca^2+^ concentration fluxes in the sub-retinal space that accompany light/dark transitions [53], [54].

Based on its *Pax6*-driven ocular expression pattern, *Trpm3/Mir204* represents an intriguing locus for eye disease. In mice, disruption of *Trpm3* results in a mild pupilary phenotype [50], whereas, *Pax6*-deficiency results in a severe eye phenotype characterized by aphakia, microphthalmia and anophthalmia [55]. The divergent nature of these eye phenotypes reflects, in part, contrasting loss-of-function effects associated with *Mir204*, *Trpm3* and *Pax6*. In *Trpm3*-null mice the gene-targeting site is located in exon-17 [49], well downstream from *Mir204* located in intron-8, and would not be expected to interfere with ocular expression of the miR-204 transcript. By contrast, early ocular expression of both *Mir204* and *Trpm3* is lost in *Pax6*-null mice [40]. Further, ocular defects resulting from *Trpm3* loss-of-function may be partially rescued because of functional redundancy with other TRP(M) channels expressed in the eye. For example, *Trpm1*, which shares ∼57% amino acid identity with *Trpm3* at the protein level, is also expressed in the ciliary body [56]. Conversely, the severe ocular defects resulting from *Pax6*-deficiency cannot be functionally rescued by other ocular transcription factors.

In humans, rare deletions involving *TRPM3* have been reported in one case of Kabuki syndrome (KS, OMIM 147920), and in a nuclear family with two cases of autism [23], [24]. In the autism family, a paternal deletion of exons 1–9 that included *MIR204* was shared by two affected sons and an unaffected daughter [24]. However, no rare variants were found in the maternal copy of *TRPM3/MIR204* in the affected sons. Since ocular abnormalities were not reported in the autism family, and we do not have any evidence of autism in the family studied here, the role of *TRPM3* in autism remains unclear. By contrast, ocular abnormalities have been found in about 30–50% of KS cases [57]. Typical ocular findings include ptosis, strabismus and/or blue sclerae, whereas, less common findings include cataract, Peters' anomaly, corneal opacities and colobomas. Again, we have no evidence of KS in the family studied here, and the importance of *TRPM3* in the etiology of KS is further obscured since the majority of KS patients have been found to harbor mutations in *MLL2* on chromosome 12q [58]. Interestingly, *PAX6* mutations primarily result in a variable pan-ocular phenotype that includes aniridia (iris hypoplasia), cataract, glaucoma, corneal clouding, foveal dysplasia, and optic nerve hypoplasia [59], [60]. In addition to cataract and glaucoma, the *TRPM3* mutation (I-to-M) discovered here was associated with a mild iris anomaly in three affected family members (V:3, V:4, V9), and in one case (V:9) anterior segment dysgenesis was also documented (Figure 1b, Table S1). Such overlap in the ocular phenotype associated with *TRPM3* and *PAX6* mutations is consistent with the close functional synergy between these genes during eye development. Our data predict that the I-to-M coding mutation in *TRPM3* affects the N-terminal translation of multiple TRPM3 isoforms (Figure 5) potentially resulting in deleterious gain-of-function effects that manifest differently to loss-of-function effects in *Trpm3*-null mice. In addition, the I-to-M mutation is located upstream of the human *MIR204* homolog hosted in intron-8 of *TRPM3* (Figure 3a) and, although it is unlikely, we cannot rule out a deleterious effect on the ocular expression of *MIR204*. Further insights regarding the genotype-phenotype complexity associated with *TRPM3* await the discovery of additional disease causing mutations associated with this cation entry channel.

## Materials and Methods

### Ethics statement

Ethical approval for this study was obtained from the Washington University Human Research Protection Office (HRPO), and written informed consent was provided by all participants prior to enrollment in accordance with the tenets of the Declaration of Helsinki, and Health Insurance Portability and Accountability Act (HIPAA) regulations.

### Family participants

A 5-generation Caucasian-American pedigree segregating cataract and glaucoma was ascertained through medical records in the Department of Ophthalmology and Visual Sciences at Washington University School of Medicine (Figure 1a). Retrospective review of medical records limited our diagnoses of cataract and glaucoma to various lens opacities requiring surgical extraction for visual rehabilitation, and significant elevation of intraocular pressure (IOP>30 mm Hg) associated with or at high risk for optic nerve damage and/or visual field loss, respectively. Blood samples were obtained from 36 family members including 25 affected, seven unaffected, two undiagnosed and two spouses (Table S1). Leukocyte genomic DNA was purified using the Gentra Puregene Blood kit (Qiagen, Valencia, CA), and quantified by absorbance at 260 nm (NanoDrop 2000, Wilmington, DE).

### SNP genotyping and linkage analysis

For genome-wide linkage analysis, genotyping was performed by means of the HumanLinkage-12 genotyping beadchip and the Infinium-II whole-genome amplification and single-base extension assay (Illumina, San Diego CA), in the Genome Technology Access Center (GTAC) at Washington University. Two-point and multipoint parametric linkage analysis was performed with Superlink (v. 1.6) and GeneHunter (v. 2.1r5), respectively, from the easyLINKAGE Plus (v. 5.08) package of programs (http://genetik.charite.de/hoffmann/easyLINKAGE/). SNP marker allele frequencies used for linkage analysis were those calculated for Caucasians by the HapMap project (http://www.hapmap.org/). A frequency of 0.001 and a penetrance of 100% were assumed for the disease allele. Multipoint computation was performed with GeneHunter in sets of 100 markers.

### Microsatellite genotyping and linkage analysis

Microsatellite markers from the National Center for Biotechnology Information (NCBI) combined Généthon, Marshfield, and deCODE genetic linkage maps (http://www.ncbi.nlm.nih.gov/genome/guide/human/) were genotyped by means of a 4200 DNA analyzer running Gene ImagIR software (Li-Cor, Lincoln, NE) as described [61]. Pedigree and haploptype data were managed using Cyrillic (v. 2.1) software (FamilyGenetix Ltd., Reading, UK), and two-point LOD scores (Z) calculated using the MLINK sub-program from the LINKAGE (5.1) package of programs (http://linkage.rockefeller.edu/soft/). Marker allele frequencies were assumed to be equal. A frequency of 0.01% and a penetrance of 100% were assumed for the disease allele.

### Whole exome and custom-target capture

Whole exome capture was achieved using the SureSelect Human All Exon 50 Mb Kit (Agilent Technologies), and custom-target capture was performed with the SureSelect Target Enrichment Kit (Agilent) and oligo-probes designed using the web-based eArray application (http://www.genomics.agilent.com/en/Custom-Design-Tools/eArray/?cid=AG-PT-122&tabId=AG-PR-1047), according to manufacturer's instructions. Briefly, genomic DNA (3 ug) was fragmented (150–200 bp) by acoustic shearing, ligated to adapter primers, and PCR-amplified. Following denaturation (95°C, 5 min), amplified DNA-fragment libraries (∼500 ng) were hybridized in solution under high stringency (65°C, 24 hr) with biotinylated RNA capture probes (∼120 bp). Resulting DNA/RNA hybrids were recovered by streptavidin-coated magnetic bead separation (Dynal, Invitrogen) and captured DNA eluted (NaOH) prior to next-generation sequencing.

### Next-generation sequencing

Solid-phase (flow-cell) massively parallel sequencing was performed on a HiSeq2000 System (Illumina) using the Illumina Multiplexing Sample Preparation Oligo-nucleotide Kit, and the HiSeq 2000 Paired-End Cluster Generation Kit according to the manufacturers instructions. Briefly, hybrid-capture libraries were amplified to add indexing (identifying) tags and sequencing primers then subject to paired-end (2×101 bp read length), multiplex sequencing-by-synthesis using fluorescent reversible (3′-blocked) terminators. Raw sequence data was aligned to the reference genome by NovoalignMPI (www.novocraft.com), and sequence variants called using the Sequence Alignment/Map format (SAMtools) and Picard programs (http://samtools.sourceforge.net/), and further annotated using SeattleSeq (http://snp.gs.washington.edu/SeattleSeqAnnotation131/). Target coverage and read depth were reviewed by the Integrated Genomics Viewer (IGV, http://www.broadinstitute.org/igv/). Variants were reviewed for presence/absence in dbSNP and 1000_genomes databases, frequency in the Exome Variant Server/database (http://evs.gs.washington.edu/EVS/), and predicted effect on protein function using the PolyPhen2 and SIFT servers [62], [63].

### Sanger Sequencing

Genomic sequence for *TRPM3* (Gene ID: 80036) was obtained from the Ensembl human genome browser (http://www.ensembl.org/index.html), and gene-specific M13-tailed PCR primers (Table S4) were selected from the NCBI re-sequencing amplicon (RSA) probe database (http://www.ncbi.nlm.nih.gov/sites/entrez?db=probe) or custom designed with Primer Quest (IDT.com) or Exon Primer (UCSC Genome Bioinformatics - http://genome.ucsc.edu). Genomic DNA (2.5 ng/ul, 20 ul reactions), was amplified (35–40 cycles) in a GeneAmp 9700 thermal cycler using AmpliTaq polymerase (Applied Biosystems, Foster City, CA) and gene-specific primers (10 pmol). Resulting PCR amplicons were either enzyme-purified with ExoSAP-IT (USB Corporation, Cleveland, OH) or gel-purified with the QIAquick gel-extraction kit (Qiagen). Purified amplicons were direct cycle-sequenced in both directions with BigDye Terminator Ready Reaction Mix (v3.1) containing M13 forward or reverse sequencing primers then ethanol precipitated and detected by capillary electrophoresis on a 3130xl Genetic Analyzer running Sequence Analysis (v5.2) software (Applied Biosystems), and Chromas (v2.23) software (Technelysium, Tewantin, Queensland, Australia).

### RT-PCR

Postmortem human lens total RNA was isolated with Trizol Reagent (Invitrogen), and reverse transcribed into cDNA by random priming with the iScript cDNA Synthesis Kit (Bio-Rad). TRPM3 sequence located between exon 1 and exon 5 was amplified with AmpliTaq polymerase (Applied Biosystems) and TRPM3 gene-specific primers (Table S5). Sequence identity was confirmed by Sanger sequencing as above. For quantization purposes, RT-PCR products were amplified in a 10-fold dilution series (in triplicate) by means of the iQ SYBR green Supermix (Bio-Rad) in a real-time PCR detection system (iQ5, Bio-Rad). Transcript copy number was determined by melt-curve analysis and standardization against a control transcript (RPL19) that was amplified separately in a similar 10-fold dilution series of the same RT-PCR products using RPL19 gene-specific forward (5′-catccgcaagcctgtgac) and reverse (5′-gtgaccttctctggcattcg) primers.

### 5′-RACE

Rapid amplification of 5′-cDNA ends was achieved using the GeneRacer Kit (Invitrogen). Briefly, total lens RNA (1–5 ug) was dephosphorylated (CIP), decapped (TAP), and ligated to the GeneRacer RNA oligo with T4 RNA ligase then reverse transcribed with AMV-RT and random primers. First-strand cDNA was PCR amplified using the GeneRacer 5′-primer and a reverse TRPM3 gene-specific primer and then re-amplified using nested primers (Table S5) to increase product specificity and yield. Following purification (SNAP column), resulting 5′-RACE products with 3′A-overhangs were sub-cloned into pCR4-TOPO and Sanger sequenced in both directions using flanking T7 and T3 sequencing primers.

### GFP fusion product

Generation of a C-terminal TRPM3-GFP fusion product was achieved using the GFP Fusion TOPO TA Expression Kit (Invitrogen) according to the manufacturer's instructions. Briefly, lens total RNA (1 ug) was reverse transcribed into cDNA by random priming with the iScript cDNA Synthesis Kit (Bio-Rad). TRPM3 coding sequence located between codons 60–154 of isoform-k (Figure 5) was PCR amplified using either a wild-type (I65) or mutant (M65) forward primer located in exon-3 paired with a reverse anchor primer located in exon-4 (Table S5). PCR product (283 bp) with non-template 3′-A overhang was ligated to the C-terminal fusion vector (pcDNA3.1/CT-GFP-TOPO) using topoisomerase. Following transformation of competent TOP10 cells, plasmid DNA was purified from overnight cultures by the QIAprep Spin Kit (Qiagen), and Sanger sequenced using the T7 and GFP-reverse sequencing primers to verify correct orientation and reading frame.

### Cell culture and transfection

HEK293T cells were cultured (37°C, 5% CO_2_) in Dulbecco's modified Eagle's medium (DMEM) supplemented with 2 mM glutamine, 100 IU penicillin, 100 ug streptomycin (Cellgro, Mediatech, Manassas, VA), and 10% bovine serum (Invitrogen). Plasmid DNA (8 ug) was transfected into HEK293T cell monolayers in 60 mm dishes (60–80% confluence) using Lipofectamine 2000 reagent (Invitrogen) in serum-free media (OptiMEM, Invitrogen) for 4 hr and then cultured for 24–48 hr in serum supplemented media. Transfected cells were washed (PBS), detached (EDTA), and centrifuged (1500×g, 5 min).

### Immunoblot analysis

Transfected cell pellets were re-suspended (50 ul, 37°C, 10 min) in detergent lysis buffer (1% IGEPAL, 50 mM Tris-HCL, 150 mM NaCl, pH 7.8; Sigma) then centrifuged (10,000×g, 10 min) to pellet cell nuclei. Post-nuclear lysate was removed and soluble protein concentration was determined using the Non-interfering assay (G-Bioscience). Soluble proteins (20 ug) were separated on SDS-PAGE gels (12%) then transferred onto nitrocellulose, incubated with anti-GFP rabbit serum (Invitrogen, dil 1∶1000), and visualized using an Odyssey Infrared Imaging System (Li-Cor Bioscience).

## Supporting Information

Figure S1 **Genome-wide linkage analysis of ocular disease in the family using SNP markers.** (**A**) Parametric two-point LOD scores (pLOD SPT) indicating linkage to chromosome 9. (**B**) Parametric multi-point LOD scores (pLOD MPT) confirming linkage to chromosome 9.
(TIF)

Figure S2 **N-terminal reading frame of the novel lens abundant TRPM3 transcript (KF987075) detected in** **Figure 4** **.** A previously identified lens EST (BM712132) [30] is shaded grey. The predicted translation initiator methionine is shown in bold. Note, the translation initiator methionine for transcript variants 1–8 is located 97 codons downstream (bold underlined). The I8 M mutation site is shown in red. Upstream translation stop-codons, in-frame with the translation start-codons, are shown in italics. Paired PCR primer sequences (Table S5) located in the EST and in exon 10 are underlined. (∧) indicates intron boundaries between the EST and exons 3, 4, 5, 6, 7, and 9 (exon 8 is skipped).
(TIFF)

Figure S3 **N-terminal coding sequence of TRPM3 that was used to generate a GFP fusion product (** **Figure 5** **).** Note the A-to-G missense change (I-to-M substitution) is embedded in a partially consensus Kozak translation start-site (TGG ATG G). Several potential out-of-frame translation start-sites are located between the predicted I-to-M substitution site (red) and the consensus Kozak start-site for GFP (A/GNN ATG G). Vector sequence is shown in italics.
(TIF)

Table S1 **Clinical ocular status of affected individuals in the family.**
(XLSX)

Table S2 **SNP haplotype for the ocular disease locus on chromosome 9.**
(DOCX)

Table S3 **Transcript variants of** ***TRPM3*** **derived from alternative splicing.**
(XLSX)

Table S4 **PCR primers for Sanger sequencing of TRPM3 exons.**
(DOCX)

Table S5 **RT-PCR primers for amplification of TRPM3 cDNA.**
(DOCX)
