# Supplementary figures and images for: Mutation of the Melastatin-Related Cation Channel, TRPM3, Underlies Inherited Cataract and Glaucoma
Source: PLoS One. 2014 Aug 4;9(8):e104000. doi: 10.1371/journal.pone.0104000 (PMC4121231; doi:10.1371/journal.pone.0104000)

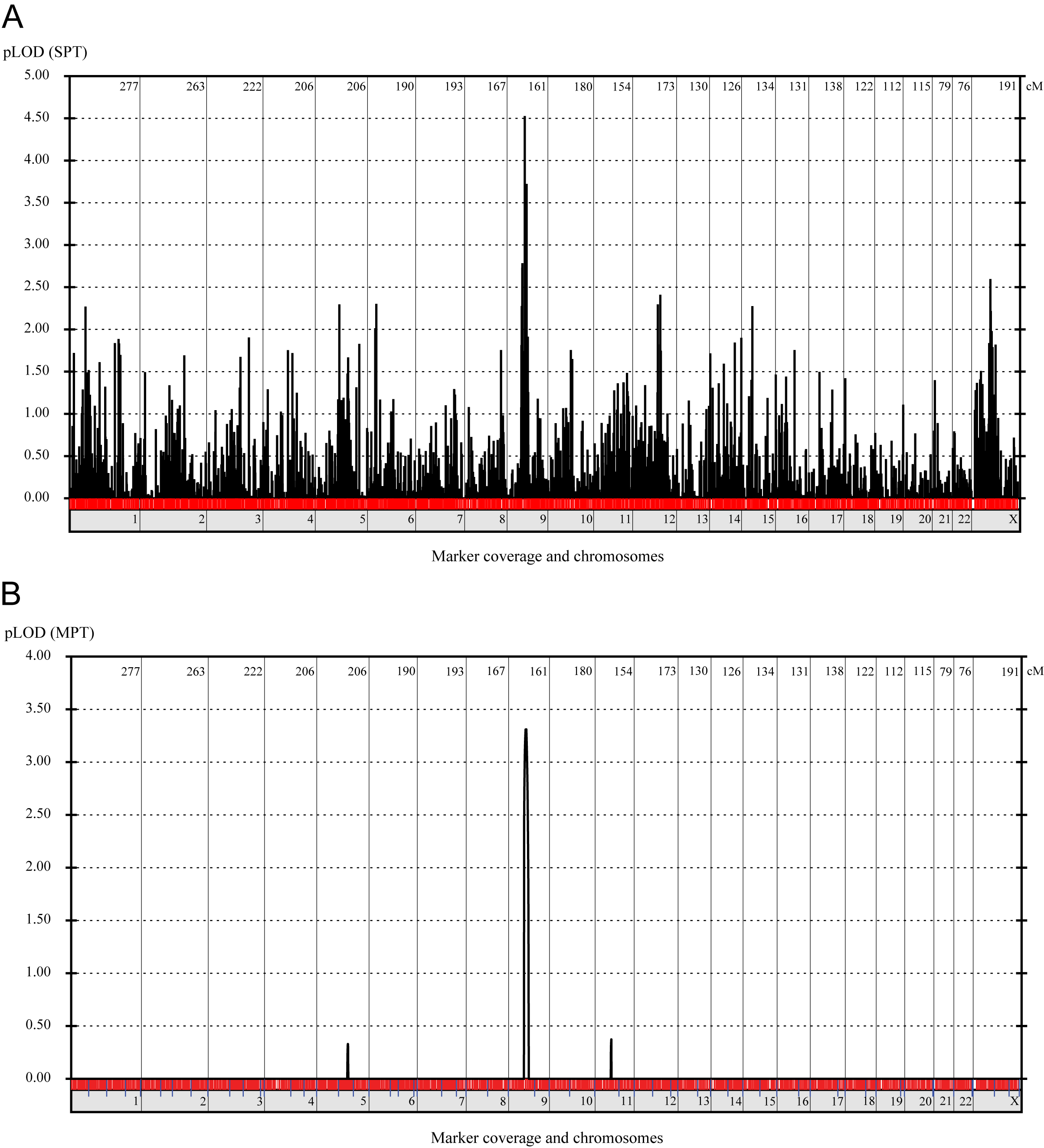

Supplement: Figure S1 — Genome-wide linkage analysis of ocular disease in the family using SNP markers. (A) Parametric two-point LOD scores (pLOD SPT) indicating linkage to chromosome 9. (B) Parametric multi-point LOD scores (pLOD MPT) confirming linkage to chromosome 9. (TIF) [file pone.0104000.s001.tif]

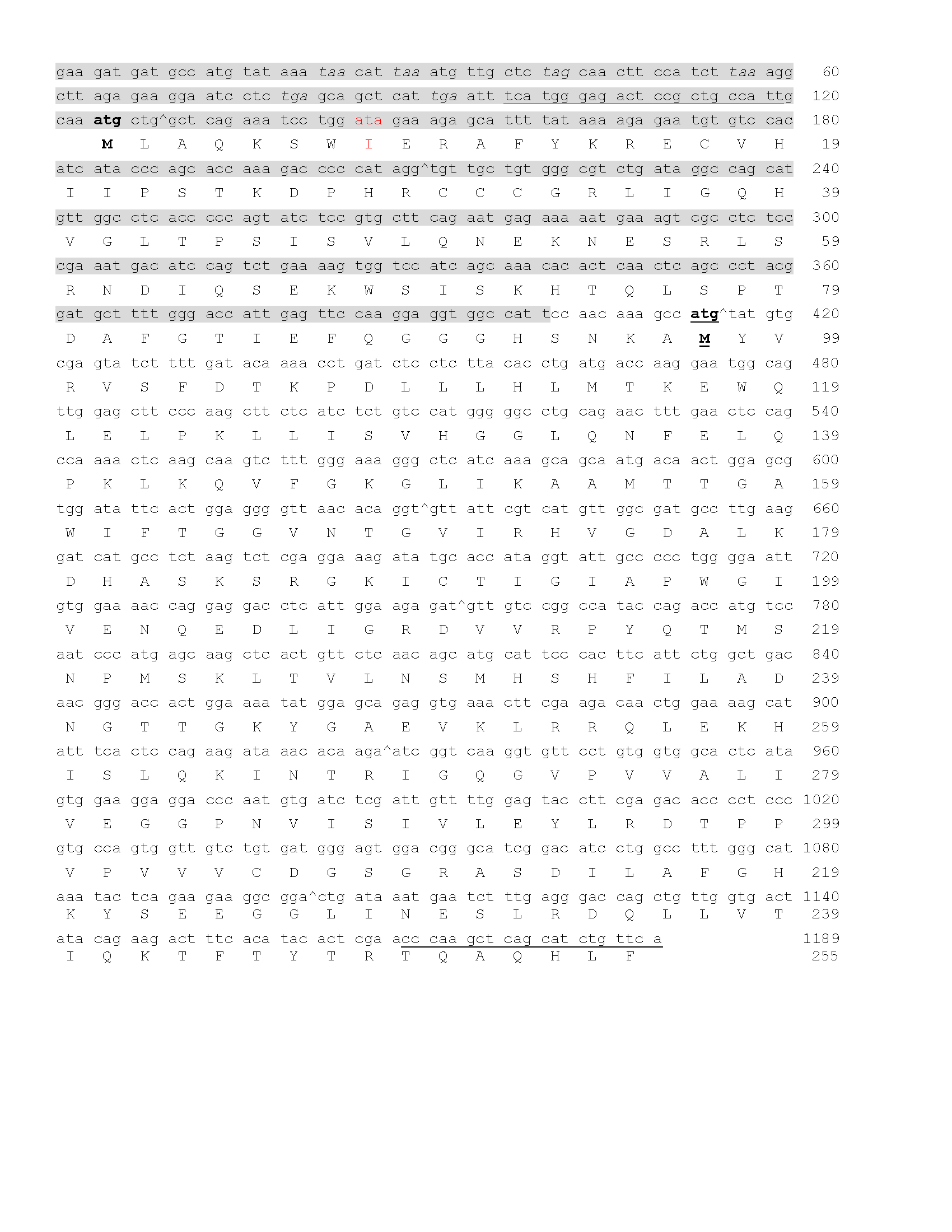

Supplement: Figure S2 — N-terminal reading frame of the novel lens abundant TRPM3 transcript (KF987075) detected in Figure 4 . A previously identified lens EST (BM712132) [30] is shaded grey. The predicted translation initiator methionine is shown in bold. Note, the translation initiator methionine for transcript variants 1–8 is located 97 codons downstream (bold underlined). The I8 M mutation site is shown in red. Upstream translation stop-codons, in-frame with the translation start-codons, are shown in italics. Paired PCR primer sequences (Table S5) located in the EST and in exon 10 are underlined. (∧) indicates intron boundaries between the EST and exons 3, 4, 5, 6, 7, and 9 (exon 8 is skipped). (TIFF) [file pone.0104000.s002.tif]

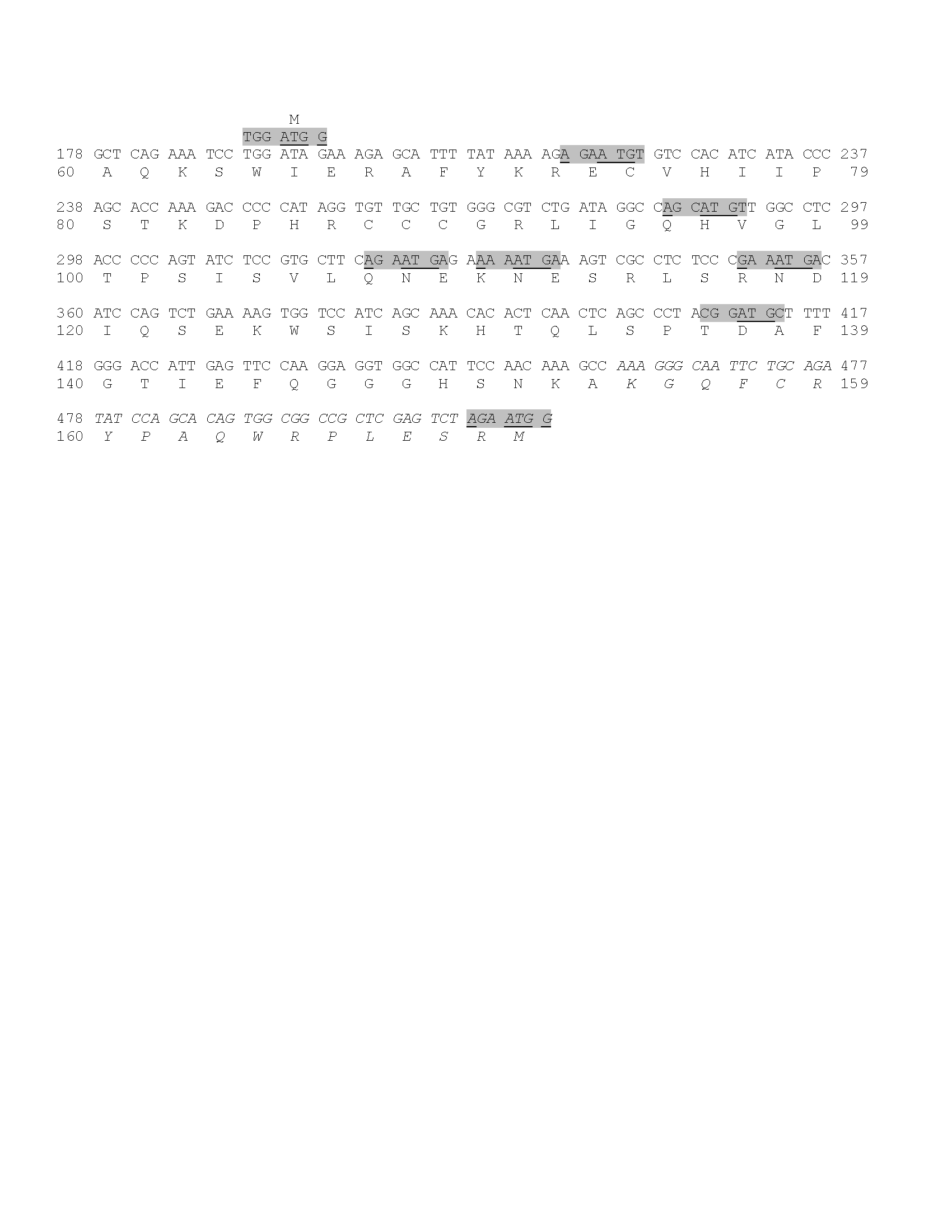

Supplement: Figure S3 — N-terminal coding sequence of TRPM3 that was used to generate a GFP fusion product ( Figure 5 ). Note the A-to-G missense change (I-to-M substitution) is embedded in a partially consensus Kozak translation start-site (TGG ATG G). Several potential out-of-frame translation start-sites are located between the predicted I-to-M substitution site (red) and the consensus Kozak start-site for GFP (A/GNN ATG G). Vector sequence is shown in italics. (TIF) [file pone.0104000.s003.tif]
