# Supplementary material for: Mutation of the Melastatin-Related Cation Channel, TRPM3, Underlies Inherited Cataract and Glaucoma
Source: PLoS One. 2014 Aug 4;9(8):e104000. doi: 10.1371/journal.pone.0104000 (PMC4121231; doi:10.1371/journal.pone.0104000)
Supplement: Table S2 — SNP haplotype for the ocular disease locus on chromosome 9. (DOCX) [file pone.0104000.s005.docx]

| Distance from  9-ptel (nt) | SNP | Alleles | Disease Allele  (recombinant) | pLOD  (θ = 0.00) |
| --- | --- | --- | --- | --- |
| 38,375,215 | rs987187 | T/C | T (V:6, C/C) | -∞ |
| 71,117,279 | rs923784 | T/C | C | 1.48 |
| 71,193,615 | rs2026349 | C/A | A | 0.42 |
| 71,456,380 | rs1414944 | G/A | G | 0.18 |
| 73,164,712 | rs1889915 | T/C | T | 1.33 |
| 74,016,773 | rs1074670 | A/G | G | 1.74 |
| 74,384,876 | rs1404195 | G/A | G | 1.17 |
| 77,168,748 | rs2031197 | C/T | T | 1.47 |
| 77,502,160 | rs1333342 | G/A | A | 4.53 |
| 79,159,993 | rs927632 | G/T | G | 1.99 |
| 79,425,889 | rs1891835 | C/T | T | 0.84 |
| 80,350,999 | rs2839899 | C/T | T | 0.24 |
| 80,428,935 | rs1887773 | G/A | G | 0.01 |
| 81,295,919 | rs722642 | T/C | T | 2.70 |
| 81,312,120 | rs713478 | G/T | G | 3.45 |
| 81,816,301 | rs735914 | G/A | A | 3.72 |
| 82,130,861 | rs1417080 | T/C | T | 0.25 |
| 84,227,158 | rs870713 | C/A | C | 0.67 |
| 84,495,227 | rs2378592 | G/A | G (V:7, AA) | -∞ |
