# Supplementary material for: Mutation of the Melastatin-Related Cation Channel, TRPM3, Underlies Inherited Cataract and Glaucoma
Source: PLoS One. 2014 Aug 4;9(8):e104000. doi: 10.1371/journal.pone.0104000 (PMC4121231; doi:10.1371/journal.pone.0104000)
Supplement: Table S4 — PCR primers for Sanger sequencing of TRPM3 exons. (DOCX) [file pone.0104000.s007.docx]

| Primer | Location | Strand | Sequence (5´- 3´) | Amplicon (bp) |
| --- | --- | --- | --- | --- |
| TRPM3 Ex1F | 5´-region | Sense | GACCTGCGTTTACTGGGAG | 561 |
| TRPM3 Ex1R | IVS 1 | Antisense | CAGCCCAAGTCCCCAAG |  |
| TRPM3 Ex2F | IVS 2 | Antisense | CCTGTCTTCATCAGAACAAACATCCC | 580 |
| TRPM3 Ex2R | IVS 1 | Sense | TCACACTTTCACAAGCTTTCTCTCTGA |  |
| TRPM3 Ex3F | IVS 3 | Antisense | GACAGTGTTTCCCTTGTAACAGGTCA | 479 |
| TRPM3 Ex3R | IVS 2 | Sense | TGCAAATAGCATTGTCTTTCTGTTCTG |  |
| TRPM3 Ex4F | IVS 4 | Antisense | CCCTTTGCAGGACTTGAGACTTGA | 521 |
| TRPM3 Ex4R | IVS 3 | Sense | GGACGGAGAAGGGCAGGAGA |  |
| TRPM3 Ex5F | IVS 5 | Antisense | GCACAACATTCCCATGGCCT | 594 |
| TRPM3 Ex5R | IVS 4 | Sense | TGTTGTACACATGTAAAGGGTGGTGAA |  |
| TRPM3 Ex6F | IVS 6 | Antisense | TGATGGGTGAAGAAAGACCACTGC | 594 |
| TRPM3 Ex6R | IVS 5 | Sense | CCAATGCATCTGGCATTACAGC |  |
| TRPM3 Ex7F | IVS 7 | Antisense | GAGCCATGCCAGTGGGAACA | 378 |
| TRPM3 Ex7R | IVS 6 | Sense | TTGCCATAAATCTTGCCTCTATCCA |  |
| TRPM3 Ex8F | IVS 8 | Antisense | TCCCAATAGTGCCGTCAAGCTG | 454 |
| TRPM3 Ex8R | IVS 7 | Sense | TGGTGCAGAAGGGCTGGATG |  |
| TRPM3 Ex9F | IVS 9 | Antisense | TTTGATTTGAGGTCTTGGTTGAGC | 532 |
| TRPM3 Ex9R | IVS 8 | Sense | CCCGTGCCTTCACTGACCC |  |
| TRPM3 Ex10F | IVS 10 | Antisense | CTGGAGCCAATGGAGCCTGA | 539 |
| TRPM3 Ex10R | IVS 9 | Sense | GGGCATTAGACACATGCCATATGAA |  |
| TRPM3 Ex11F | IVS 10 | Sense | TTATGTGTGCTTACCCTGTGG | 352 |
| TRPM3 Ex11R | IVS 11 | Antisense | TCTTACAGGAGAGACCCCTATG |  |
| TRPM3 Ex12F | IVS 12 | Antisense | CCACTAGATGGCGGTGCTTTCC | 407 |
| TRPM3 Ex12R | IVS 11 | Sense | GCCAAGCAGGCTCTTTGGGA |  |
| TRPM3 Ex13F | IVS 13 | Antisense | GCGGAATCTTGGTGACTTGGTG | 491 |
| TRPM3 Ex13R | IVS 12 | Sense | TGGGAGAGGTGTGGCTGTCC |  |
| TRPM3 Ex14F | IVS 14 | Antisense | TCTGAAATGAGTGGCGCCTGA | 312 |
| TRPM3 Ex14R | IVS 13 | Sense | CACTGAGCAACTAGAACAAGGTCCATC |  |
| TRPM3 Ex16F | IVS 16 | Antisense | TTGTTTAGGTTCACTCTCAGCGCA | 597 |
| TRPM3 Ex15R | IVS 14 | Sense | GCATTTGCCCTGTCTAGCCTTG |  |
| TRPM3 Ex17F | IVS 17 | Antisense | TGCTGTGAGCAACCCTTATGACA | 545 |
| TRPM3 Ex17R | IVS 16 | Sense | GCCAAGCGTGGTAACATGCAC |  |
| TRPM3 Ex18F | IVS 18 | Antisense | TGTGTCCATCCTGTGTGTGCATC | 555 |
| TRPM3 Ex18R | IVS 17 | Sense | GGACAGGAGCTGGCCTCGTT |  |
| TRPM3 Ex19F | IVS 19 | Antisense | GCTAATGCAGCAGGACCCAGG | 517 |
| TRPM3 Ex19R | IVS 18 | Sense | TTGGCCAGCAGGTCCACTTT |  |
| TRPM3 Ex20F | IVS 20 | Antisense | CCTGGTGGAAGGCTGGGAAC | 377 |
| TRPM3 Ex20R | IVS 19 | Sense | CCCATACCTCAGGCTCTCTGATTC |  |
| TRPM3 Ex21F | IVS 21 | Antisense | TGTGGCCTGTGAAAGCTGCC | 537 |
| TRPM3 Ex21R | IVS 20 | Sense | CACCCAAGGCCTTCCATCTCA |  |
| TRPM3 Ex22F | IVS 22 | Antisense | CCCAGGTTTGTCAGAGGAGCAA | 531 |
| TRPM3 Ex22R | IVS 21 | Sense | TTTAAACGAAGCACCAGAGCCC |  |
| TRPM3 Ex23F1 | Exon 23 | Antisense | GGCCCAAATACTTGTTCACGCC | 307 |
| TRPM3 Ex23R1 | IVS 22 | Sense | CCAACAGACCAAGCACTTCCCA |  |
| TRPM3 Ex23F2 | IVS 23 | Antisense | TCCCTCCCTCTCTCTCCCATCA | 568 |
| TRPM3 Ex23R2 | Exon 23 | Sense | TGTCAGAGCCAGGGAAGTTGC |  |
| TRPM3 Ex24F | IVS 24 | Antisense | CCAGATGTCGATTCGTGTCACAG | 590 |
| TRPM3 Ex24R | IVS 23 | Sense | CACTGGCTTCAACAGACCAAGCA |  |
| TRPM3 Ex25F | IVS 25 | Antisense | GGCAGAACTGGCCTTTCATGG | 557 |
| TRPM3 Ex25R | IVS 24 | Sense | CCACCACCACAGTAGCAGCCC |  |
| TRPM3 Ex26F | IVS 26 | Antisense | CCAAGCCTTCAGAGCTGTCTGC | 558 |
| TRPM3 Ex26R | IVS 25 | Sense | ATCCAGCTGCCTCCCTGCAA |  |
| TRPM3 Ex27F | IVS 27 | Antisense | CTGAGCCAGCAACCTCAGGC | 573 |
| TRPM3 Ex27R | IVS 26 | Sense | GCAAGTTGCCTAATTGGATTTGGA |  |
| TRPM3 Ex28F1 | Exon 28 | Antisense | GGCTGCAGGAGCTTTGGGTT | 580 |
| TRPM3 Ex28R1 | IVS 27 | Sense | TGAAGGTACCAAGGTCAGGCACA |  |
| TRPM3 Ex28F2 | Exon 28 | Antisense | GGTGTGGTGGCTAGGTAGCGG | 578 |
| TRPM3 Ex28R2 | Exon 28 | Sense | CCCTGCAGGTGAGGAGACCA |  |
| TRPM3 Ex28F3 | Exon 28 | Antisense | GGCGCGCTCTATCTTGGGAA | 570 |
| TRPM3 Ex28R3 | Exon 28 | Sense | TGCAACACTTGCACCCACAGA |  |
| TRPM3 Ex28F4 | 3´-region | Antisense | TGGCCCAGAAGTCACCTTTGA | 596 |
| TRPM3 Ex28R4 | Exon 28 | Sense | CGGACAGAGCTGCCTTCCCT |  |
