# Supplementary material for: Mutation of the Melastatin-Related Cation Channel, TRPM3, Underlies Inherited Cataract and Glaucoma
Source: PLoS One. 2014 Aug 4;9(8):e104000. doi: 10.1371/journal.pone.0104000 (PMC4121231; doi:10.1371/journal.pone.0104000)
Supplement: Table S5 — RT-PCR primers for amplification of TRPM3 cDNA. (DOCX) [file pone.0104000.s008.docx]

| Primer | Use | Location | Strand | Sequence (5´- 3´) |
| --- | --- | --- | --- | --- |
| TRPM3 utRefF | GFP-fusion | Exon 3 | Sense | GCTCAGAAATCCTGGATAGAAAGAGCA |
| TRPM3 utMutF | GFP-fusion | Exon 3 | Sense | GCTCAGAAATCCTGGATGGAAAGAGCA |
| TRPM3 utR | GFP-fusion | Exon 4 | Antisense | TGGCTTTGTTGGAATGGCCACC |
| TRPM3 RT6R | 5´-RACE, RT-PCR????? | Exon 6 | Antisense | TCAAGGCATCGCCAACATGACGA |
| TRPM3 RT7R | 5´-RACE | Exon 7 | Antisense | CCAGTGGTCCCGTTGTCAGCC |
| TRPM3 RT1F | RT-PCR | Exon 1 | Sense | AGCTGTGCCACGCAGCCTTT |
| TRPM3 RT2F | RT-PCR | Exon 2 | Sense | AGCTGCTCCGGGGACTGCTT |
| TRPM3 RT3F | RT-PCR | Exon 3 | Sense | TGTGTCCACATCATACCCAGCACC |
| TRPM3 ESTF | RT-PCR | BM_712132 (EST) | Sense | TCATGGGAGACTCCGCTGCCATT |
| TRPM3 RT5R | RT-PCR | Exon 5 | Antisense | GCTTGGGAAGCTCCAACTGCCAT |
| TRPM3RT10R | RT-PCR | Exon 10 | Antisense | TGAACAGATGCTGAGCTTGGG |
